# Enhanced Piezoelectricity and Thermal Stability of Electrostrain Performance in BiFeO_3_-Based Lead-Free Ceramics

**DOI:** 10.3390/nano13050942

**Published:** 2023-03-05

**Authors:** Hongwei Shi, Kai Li, Feng Li, Jianxing Ma, Yubing Tu, Mingsheng Long, Yilin Lu, Weiping Gong, Chunchang Wang, Lei Shan

**Affiliations:** 1 Information Materials and Intelligent Sensing Laboratory of Anhui Province, Anhui University, Hefei 230601, China; 2 Key Laboratory of Structure and Functional Regulation of Hybrid Materials of Ministry of Education, Anhui University, Hefei 230601, China; 3 Institutes of Physical Science and Information Technology, Anhui University, Hefei 230601, China; 4 Guangdong Provincial Key Laboratory of Electronic Functional Materials and Devices, Huizhou University, Huizhou 516001, China; 5 Laboratory of Dielectric Functional Materials, School of Materials Science & Engineering, Anhui University, Hefei 230601, China

**Keywords:** BiFeO_3_–BaTiO_3_, piezoelectricity, electrostrain, temperature stability, domain structure

## Abstract

BiFeO_3_–based ceramics possess an advantage over large spontaneous polarization and high Curie temperature, and are thus widely explored in the field of high–temperature lead–free piezoelectrics and actuators. However, poor piezoelectricity/resistivity and thermal stability of electrostrain make them less competitive. To address this problem, (1 − *x*) (0.65BiFeO_3_–0.35BaTiO_3_)–*x*La_0.5_Na_0.5_TiO_3_ (BF–BT–*x*LNT) systems are designed in this work. It is found that piezoelectricity is significantly improved with LNT addition, which is contributed by the phase boundary effect of rhombohedral and pseudocubic phase coexistence. The small–signal and large–signal piezoelectric coefficient (*d*_33_ and $d_{33}^{*}$) peaks at *x* = 0.02 with 97 pC/N and 303 pm/V, respectively. The relaxor property and resistivity are enhanced as well. This is verified by Rietveld refinement, dielectric/impedance spectroscopy and piezoelectric force microscopy (PFM) technique. Interestingly, a good thermal stability of electrostrain is obtained at *x* = 0.04 composition with fluctuation *η* = 31% ($\frac{S_{\max}^{'}-S_{\mathrm{RT}}}{S_{\mathrm{RT}}}\times 100\%$), in a wide temperature range of 25–180 °C, which is considered as a compromise of negative temperature dependent electrostrain for relaxors and the positive one for ferroelectric matrix. This work provides an implication for designing high–temperature piezoelectrics and stable electrostrain materials.

## 1. Introduction

As a mechanical–electricity conversion functional material, piezoelectric ceramics have wide applications in the defense, industrial, and medical fields, etc. [1,2,3]. Due to extraordinary piezoelectric properties and electromechanical coupling effects near morphotropic phase boundary (MPB), Pb(Zr, Ti)O_3_–based (PZT) ceramics have been a hot research topic [4,5]. However, with an increasingly serious environmental concern, lead–based materials are gradually replaced by lead–free materials. A series of lead–free materials such as (Bi_0.5_Na_0.5_)TiO_3_ (BNT–) [6,7,8], BiFeO_3_ (BF–) [9,10,11], (K_0.5_Na_0.5_)NbO_3_ (KNN–) based [12,13] ceramics have been widely developed. For example, Liu et al. reported a non-textured BNT–based ceramic with a large electrostrain value of ~0.7% at room temperature (RT), but it exhibited relatively large strain hysteresis and a decrease in electrostrain as temperature increased [14]; Due to polymorphic phase transition effect in KNN–based ceramics, the macroscopic performance usually exhibited a temperature sensitivity, although compositionally graded multilayer composite and layered distribution of dopants strategy largely alleviated this shortcoming [15,16]. In contrast, BiFeO_3_–BaTiO_3_–based (BF–BT) ceramics show a positive temperature dependence of electrostrain as their high depolarization temperature (*T*_d_) [17]. According to first–principles calculations, high spontaneous polarization and electrostrain are facilitated by the inverse rotation of oxygen octahedron and bismuth and oxygen hybridization as a result of the huge displacement of Bi^3+^ and Fe^3+^ ions [18,19]. However, a high leakage current and oxygen vacancies ($V_{o}^{¨}$) concentration is detrimental to electrical performance [20]. Zhao et al. effectively reduced $V_{o}^{¨}$ by annealing ceramics in an oxygen atmosphere [21]. In addition, electrostrain of BF–BT–based ceramics at high temperature are frequently reported. For example, Zheng et al. reported that the unipolar strain increased from 0.10% at RT to 0.32% at 200 °C for Bi(Mg_2/3_Nb_1/3_)O_3_ modified BF–BT ceramics [22]. Similarly, an electrostrain of Sm–doped BF–BT ceramics increased from 0.28% at RT to 0.52% at 125 °C [23]. Although a large electrostrain was obtained at an elevated temperature, the temperature-sensitive electrostrain performance is unfavorable for practical applications.

Similar to the thermal fluctuation effect, structural and charge disorder in relaxor ferroelectrics usually produce a negative temperature dependence of electrostrain [24,25]. Also, an increased concentration of nanodomains in relaxors reduces the domain wall energy to favor ferroelectric domains switching, which can contribute to a significant increase in electrostrain [26,27]. Therefore, to obtain electrostrain with high–temperature stability, we combine the negative temperature stability of relaxors and positive temperature stability of BF–BT ferroelectric matrix. Also, an end-member La_0.5_Na_0.5_TiO_3_ is reported to not only increase the relaxor degree, but also reduce dielectric loss [28,29]. Interestingly, phase and domain structures of BF–BT ceramics are significantly modulated by adding La_0.5_Na_0.5_TiO_3_ in this work, and high piezoelectricity/resistivity and thermal stability of electrostrain are obtained. The underlying mechanism is comprehensively analyzed by Rietveld refinement, Raman, dielectric/impedance spectroscopy, and PFM technique.

## 2. Materials and Methods

We obtained (1 − *x*) (0.65BiFeO_3_–0.35BaTiO_3_)–*x*La_0.5_Na_0.5_TiO_3_ (BF–BT–*x*LNT, *x* = 0~0.06) ceramics by conventional solid–state synthesis using Bi_2_O_3_, Fe_2_O_3_, TiO_2_, BaCO_3_, La_2_O_3,_ and Na_2_CO_3_. Excessive 2%mol Bi_2_O_3_ was added for compensation. All raw materials were mixed with alcohol and ball-milled for 15 h and then calcined at 750 °C for 6 h twice. Before the second ball-milling step, 1% mol MnO_2_ was added to reduce the leakage current. Then, powders were mixed with 8 wt.% PVA binder and pressed into the discs with a diameter of 10 mm. After removing the binder, the discs were sintered at 1040 °C for 3 h. For electrical measurements, a silver paste was coated on both sides of the polished samples and fired at 560 °C for 10 min.

The crystal structure was measured by X-ray diffractometer (XRD, Rigaku Smart–lab). A scanning electron microscope (SEM, Regulus 8230; Hitachi Co., Tokyo, Japan) was used to observe the sample microstructures. Before observing the microstructure, all sample surfaces are polished smooth and hot corroded at 950 °C for 30 min. A Raman microscope was used to acquire Raman spectra (Horiba Jobin–Yvon HR800, France). Dielectric/impedance properties were acquired by Wayne Kerr 6500B impedance analyzer (Wayne Kerr Electronic Instrument Co., Shenzhen, China). Polarization hysteresis (*P–E*) loops and electrostrain (*S–E*) curves were measured using a ferroelectric measuring system (Precision LC, Radiant Technologies, Inc. Albuquerque, NM, USA) at a frequency of 1 Hz. The sample was poled for 15 min under an electric field (*E* = 60 kV/cm) at 120 °C, and then piezoelectric coefficients *d*_33_ were measured with a quasi-static *d*_33_ m (YE2730A, China). The domain structure was characterized by the PFM technique (Asylum Research).

## 3. Results and Discussion

**Figure 1:**
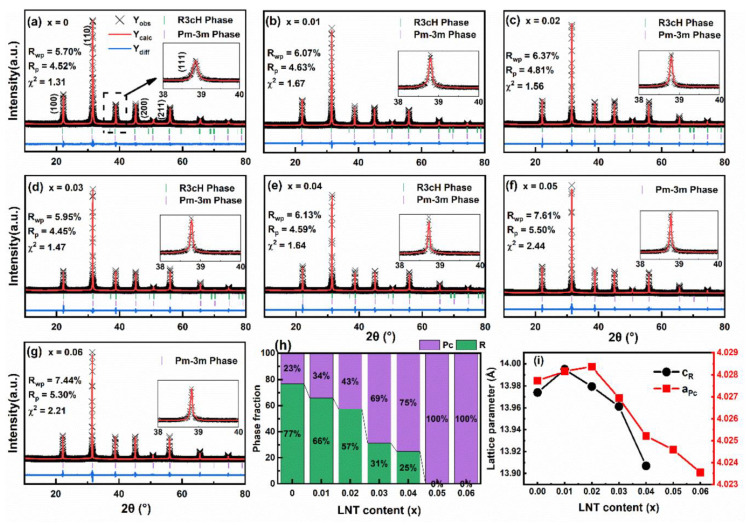
(**a**–**g**) Rietveld refinement patterns of BF–BT–*x*LNT ceramics (*x* = 0~0.06); (**h**) *R*3*cH* (*R*) and *Pm*$\bar{3}$*m* (*Pc*) phase fraction evolution and (**i**) lattice parameter of *R* and *Pc* phase as a function of LNT content.

Room temperature (RT) XRD patterns of BF–BT–*x*LNT ceramics (*x* = 0~0.06) are shown in Figure 1a–g, respectively. All samples have a pure perovskite structure without a second phase, indicating that LNT is completely dissolved into BF–BT matrix and forms a solid solution. To get an in-depth understanding of phase structure and content evolution with changing LNT, Rietveld refinement is performed and *R*3*cH* (*R* phase) and *Pm*$\bar{3}$*m* (*Pc* phase) space group models are exploited [30,31]. Low fitted values of *R*_wp_, *R*_p,_ and *χ*^2^ indicate that fitting results are reliable. Also, locally magnified (111) diffraction peaks are displayed in their insets. Obviously, a wide (111) peak for the *x* = 0 sample indicates the existence of the *R* phase. The (111) peak gradually becomes narrow and sharp with an increase in LNT content, indicating that the *R* phase is gradually substituted by a *Pc* phase, and finally evolves into a single *Pc* phase at *x* = 0.05, as displayed in Figure 1h. The refined lattice parameter is plotted in Figure 1i. Table 1 also shows the lattice parameters and *R*–factors obtained by Rietveld refinement for better understanding. Obviously, with LNT addition, the lattice parameter generally exhibits a downward shift, which is mainly attributed to smaller ionic radii of Na^+^ and La^3+^ (CN = 12, R_Na_^+^ = 1.39 Å, R_La_^3+^ = 1.36 Å) than that of Ba^2+^ and Bi^3+^ (CN = 12, R_Ba_^2+^ = 1.61 Å, R_Bi_^3+^ = 1.45 Å) [32].

**Figure 2:**
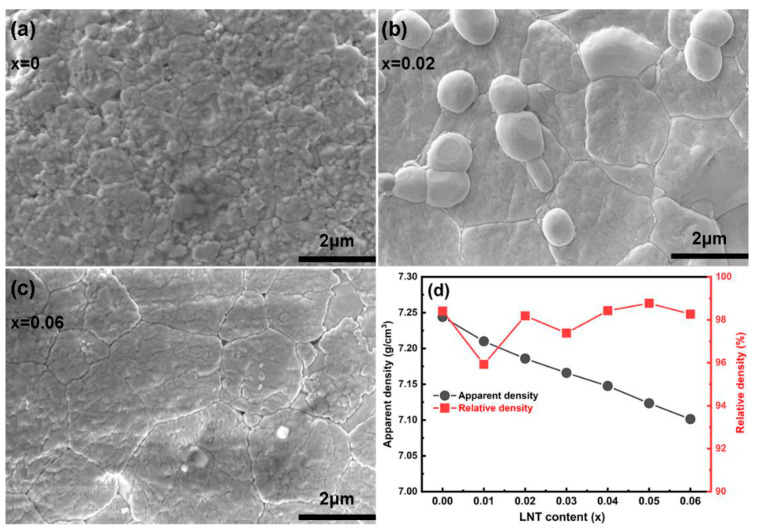
(**a**–**c**) SEM images of selected BF–BT–*x*LNT ceramics (*x* = 0, 0.02, 0.06); (**d**) apparent and relative density as a function of LNT content.

SEM images of selected compositions (*x* = 0, 0.02, 0.06) are displayed in Figure 2a–c. Clearly, all ceramic grains are uniformly distributed, and there are no obvious pores, indicating a dense ceramic microstructure. The apparent density gradually decreases with the addition of LNT, which is determined by a smaller molecular mass of LNT as compared to BF. The relative density in all compositions surpasses ~95%, which verifies their high compactness.

**Figure 3:**
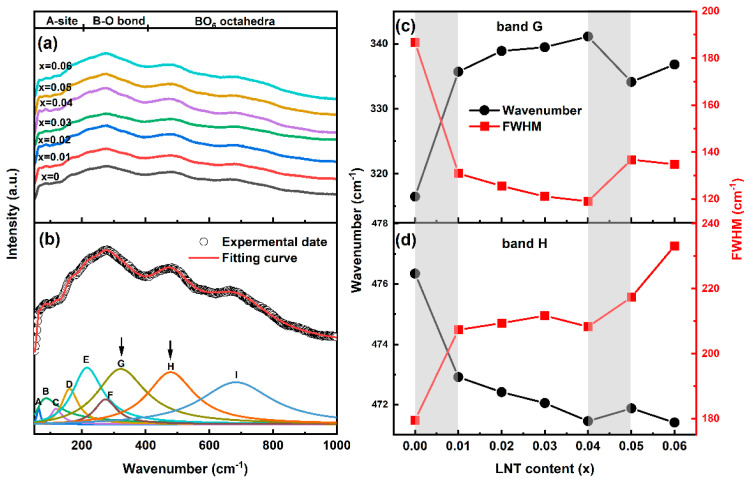
(**a**) Raman spectra of BF–BT–*x*LNT ceramics (*x* = 0~0.06); (**b**) experimental data, peak fitting curve, and the deconvolution of Raman spectra (selecting *x* = 0 composition as an example); (**c**,**d**) composition dependence of wavenumber and FWHM for selected G and H bands.

Raman spectra technique is a powerful tool to detect phase transition at short-range scales, and Raman spectra for BF–BT–*x*LNT ceramics are performed, as shown in Figure 3a. Generally, the wavenumber range in 50~1000 cm^−1^ is divided into three vibrational modes, namely A–site (50~200 cm^−1^), B–O bond (200~400 cm^−1^), and BO_6_ octahedral vibration (400~1000 cm^−1^) [33,34]. To clearly demonstrate vibrational mode changes, raw Raman spectra are fitted by the Lorentzian function, and a series of deconvoluted Raman peaks are delineated, as shown in Figure 3b. Here, two representative peaks (G and H bands) are selected to analyze the wavenumber and FWHM (full width at half maximum) evolution to detect phase transition, as shown in Figure 3c,d. Notably, two discontinuous changes in wavenumber and FWHM are observed (as highlighted by shadow), which strongly suggests that phase transition occurs [35]. Since the Raman shift is interrelated to crystal stress and polarization, the first abrupt change of G and H bands probably corresponds to distorted local stress and the polarization field [36]. With an increase of heterovalent ionic proportion (Na^+^ and La^3+^) and corresponding local random field, the second vibration change may be related to the ferroelectric-to-relaxor (FR) phase transition, which will be discussed infra in detail.

**Figure 4:**
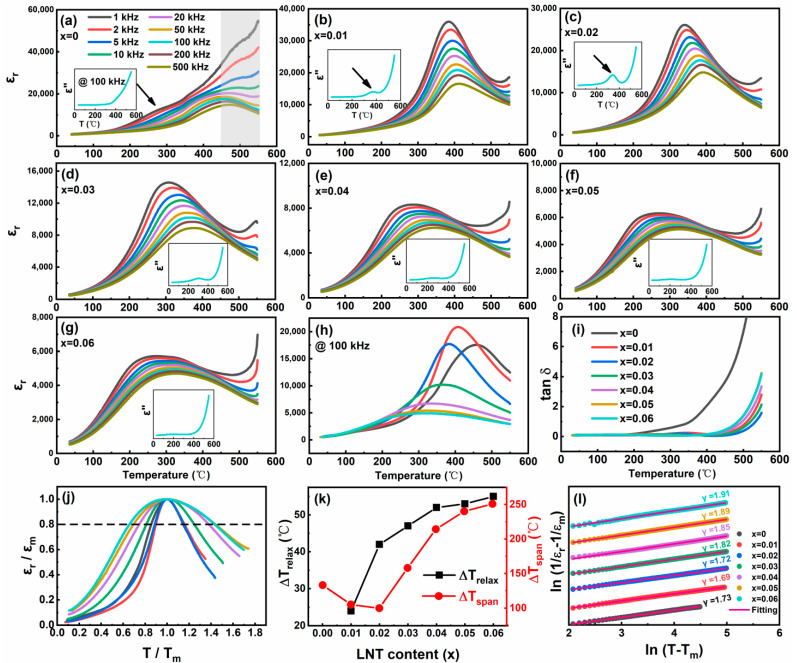
(**a**–**g**) Temperature dependence of *ε*_r_ of fresh BF–BT–*x*LNT (*x* = 0~0.06) ceramics at 1 kHz ~500 kHz, the inset shows the *ε*′′ at 100 kHz; (**h**,**i**) collection of *ε*_r_ and *tanδ* curves; (**j**) summary of *ε*_r_/*ε*_m_ versus *T*/*T*_m_; (**k**) Δ*T*_relax_, Δ*T*_span_ and (**l**) variation of *γ* at 100 kHz as a function of LNT content.

Temperature dependence of dielectric constant (*ε*_r_) of BF–BT–*x*LNT ceramics (*x* = 0~0.06) at 1 kHz~500 kHz are shown in Figure 4a–g, respectively. For *x* = 0 compositions, it shows a relaxor-like behavior near 300 °C, which is related to a dipolar relaxation caused by $V_{o}^{¨}$ hopping [37]. The dielectric drift at high temperatures is caused by a large conductivity due to an increased $V_{o}^{¨}$ motion, as highlighted in Figure 4a [38]. As *x* increases to 0.01 and 0.02, the dielectric peak becomes sharp, and *ε*_r_ at *T*_m_ (the temperature for dielectric maxima) also increases (Figure 4h). This is also observed from an obvious hump at the imaginary part of the dielectric constant (*ε*′′) curve, as shown in the insets of Figure 4b,c. Notably, the high–temperature dielectric drift is significantly suppressed, indicating the resistivity is markedly improved with LNT addition, which is also observed from the dielectric loss (*tanδ*) in Figure 4i. As to *x* = 0.03~0.06, diffusive phase transition and frequency dispersion are clearly observed, exhibiting a relaxation property [39,40]. The dielectric curves for *x* = 0~0.06 samples at 100 kHz are collected in Figure 4h. The peak *ε*_r_ value peaks at *x* = 0.01 and 0.02 and then sharply decreases for further increasing LNT content, accompanied by a wide and diffuse dielectric shape. This is more clearly indicated by *ε*_r_/*ε*_m_ versus *T*/*T*_m_ curves in Figure 4j. Therefore, an addition of an LNT component first improves the dielectric performance with an appropriate proportion of *R* and *Pc* phase. With an excessive LNT content, *ε*_r_ is significantly suppressed with a dominant relaxor *Pc* phase. In order to quantitatively describe relaxor properties for this system, two parameters of Δ*T*_relax_ [*T*_m_(100 kHz) − *T*_m_(1 kHz)] and Δ*T*_span_ (temperature span corresponds to *ε*_r_/*ε*_m_ = 0.8 in Figure 4j) are adopted [41]. Obviously, Δ*T*_relax_ and Δ*T*_span_ exhibit an increasing trend as LNT content increases, as plotted in Figure 4k. Also, a modified Curie–Weiss law is used to denote the relaxor degree *γ*: $\frac{1}{\varepsilon_{r}}-\frac{1}{\varepsilon_{m}}=\frac{{\left(T-T_{m}\right)}^{\gamma }}{C}\;\left(1\leq \gamma \leq 2\right)$, where *C* is Curie–Weiss constant, *ε*_m_ is dielectric maxima and the parameter *γ* is adopted to reveal the relaxor degree [42,43]. As shown in Figure 4l, *γ* value steadily increases from 1.73 for *x* = 0 to 1.91 for *x* = 0.06 composition. Therefore, an enhanced relaxor properties are obtained with LNT addition.

**Figure 5:**
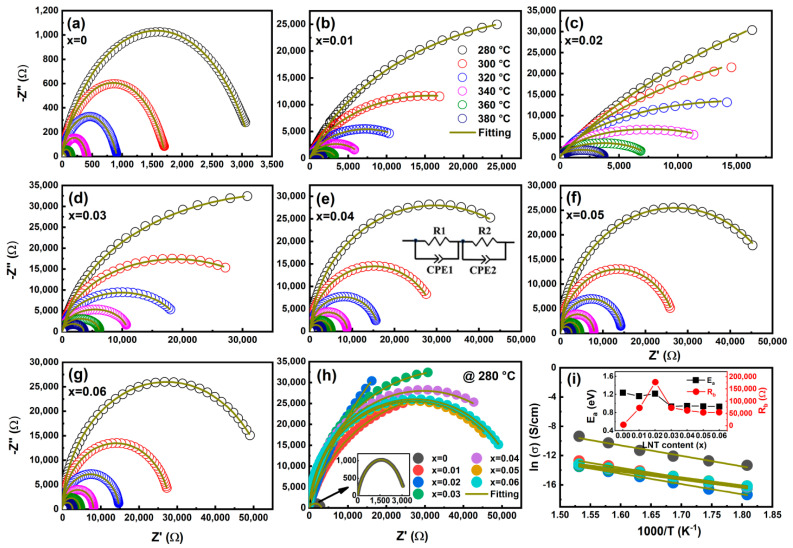
(**a**–**g**) Complex impedance plots for BF–BT–*x*LNT (*x* = 0~0.06) ceramics in the temperature range of 280–380 °C with an interval of 20 °C, the inset of (**e**) exhibits the fitting electric circuit; (**h**) complex impedance spectra for *x* = 0~0.06 samples at 280 °C; (**i**) ln*σ* with inverse of temperature (1000/*T*), the inset shows the variation of *E*_a_ and *R*_b_ as a function of LNT content.

To determine the resistivity and conduction mechanism for BF–BT–*x*LNT ceramics, complex impedance spectra (CIS) are measured over 280–380 °C with an interval of 20 °C, as shown in Figure 5a–g. All ceramics exhibit a single semicircle complex impedance shape, which is related to a single relaxation mechanism of bulk response [44]. The data are well fitted by two parallel R–CPE equivalent circuits, as shown in the inset of Figure 5e, which represent the grain boundary and bulk (grain) contributions, respectively. As known, bulk resistance (*R*_b_) often dominates ferroelectric ceramics, and an extrapolated intercept on $Z^{\prime }$ axis corresponds to *R*_b_ in CIS. Obviously, *R*_b_ gradually decreases with increasing temperature, indicating a negative temperature coefficient behavior. The *R*_b_ value is obtained by fitting the experimental CIS, as shown in Figure 5a–g. For a better understanding, the *R*_b_ values are also listed in Table 2. Interestingly, the LNT addition greatly increases the *R*_b_ of the BF–BT matrix (Figure 5h,i), which is consistent with a depressed *tanδ*. In addition, bulk conductivity (*σ*_b_) and activation energy (*E*_a_) are calculated by the following formulas [45]:

(1) $$\sigma_{b}=\frac{h}{S\times R_{b}}$$

(2) $$\sigma_{b}=\sigma_{0}\exp\left(-\frac{E_{a}}{k_{B}T}\right)$$

where *h* is sample thickness, *S* is electrode area, *k*_B_ is Boltzmann constant, and *T* is the measured temperature. Figure 5i depicts the *σ*_b_ as a function of inverse of temperature, and the fitted value of *E*_a_ is obtained between 0.93 and 1.24 eV, which is close to the *E*_a_ of $V_{o}^{¨}$ (~1 eV) [46]. Therefore, $V_{o}^{¨}$ dominates the high–temperature conductivity and leads to a large leakage current, which is mainly due to the volatilization of bismuth and the reduction of Fe^3+^ [47].

**Figure 6:**
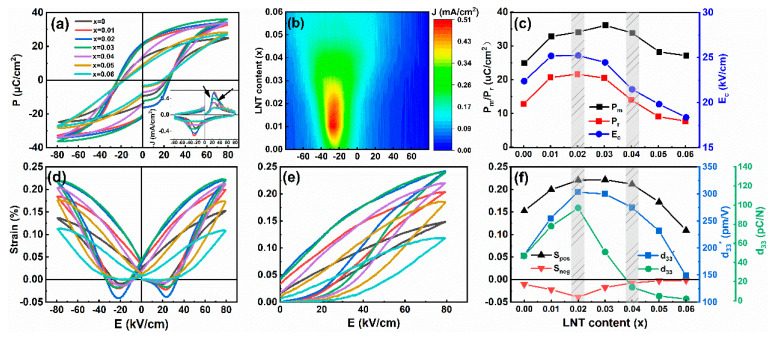
(**a**) RT *P*–*E* loops for BF–BT–*x*LNT (*x* = 0~0.06) ceramics at 1 Hz, the inset shows *J*–*E* loops; (**b**) the contour map of *J*–*E* value; (**c**) evolution of *P*_m_, *P*_r_ and *E*_c_ as a function of LNT content; (**d**) bipolar and (**e**) unipolar *S*–*E* curves; (**f**) *S*_pos_, *S*_neg_, *d*_33_ *, and *d*_33_ as a function of LNT content.

Polarization hysteresis (*P–E*) and corresponding current density (*J–E*) loops of BF–BT–*x*LNT ceramics at RT are measured at 1 Hz, as shown in Figure 6a,b. Low saturated/remanent polarization (*P*_m_/*P*_r_) with an incomplete domain switching is observed for *x* = 0 composition. Interestingly, for *x* = 0.01 and 0.02 samples, saturated *P*–*E* loops and a sharp *J*–*E* peak are observed, featuring a normal ferroelectric. A sharp *J*–*E* peak usually indicates strong ferroelectricity with a fast domain switching behavior under *E* [26]. As to *x* = 0.03 and 0.04, splitting *J*–*E* peaks are observed, indicating an emergence of an intermediate state with nonergodic/ergodic and ferroelectric phase coexistence. Meanwhile, *P*_m_, *P*_r,_ and coercive field (*E*_c_) decrease strikingly, as plotted in Figure 6c. Slant *P–E* loops and *J–E* platform with low *P*_r_ and *E*_c_ value indicates a pure relaxor phase for *x* = 0.05 and 0.06 samples, which also accords with pure *Pc* phase for both compositions. In addition, bipolar and unipolar electrostrain (*S*–*E*) curves of BF–BT–*x*LNT ceramics are shown in Figure 6d,e, respectively. For *x* ≤ 0.03 samples, typical butterfly-shape bipolar *S*–*E* curves are present, and they gradually evolve into a sprout–shape [decrease in negative strain (*S*_neg_)] with increasing LNT content. In general, larger *S*_neg_ indicates more non-180° domain switching under *E*, and this predicts improvement of piezoelectric performance. For *x* = 0.04~0.06 samples, an almost zero–*S*_neg_ indicates a gradual FR phase transition. Clearly, positive strain (*S*_pos_) and *S*_neg_ peaks *x* = 0.02 sample, and the normalized $d_{33}^{*}$ ($S_{\max}/E_{\max}$) is calculated as well, as plotted in Figure 6f. The *d*_33_ and $d_{33}^{*}$ value simultaneously achieve optimal value (97 pC/N and 303 pm/V) at *x* = 0.02, which is contributed by phase boundary effect with proper proportion of *R* and *Pc* phase content. Excessive addition of LNT degrades piezoelectricity and ferroelectricity and thus $d_{33}^{*}$ decreases.

**Figure 7:**
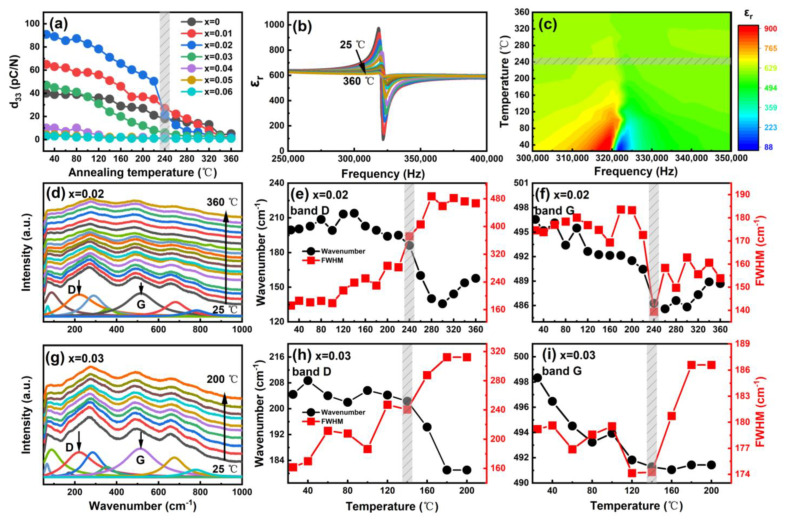
(**a**) Variation of *d*_33_ with thermal annealing at various temperatures for BF–BT–*x*LNT ceramics; (**b**,**c**) frequency dependent *ε*_r_ evolution for poled *x* = 0.02 sample at different annealing temperature and corresponding contour plot; (**d**,**g**) temperature dependent Raman spectra in 25–360 °C and (**e**,**f**,**h**,**i**) evolution of wavenumber and FWHM for selected D and G modes for *x* = 0.02 and 0.03 samples.

To measure the temperature stability of piezoelectric performance for these compositions, *d*_33_ is collected at elevated annealing temperature, as shown in Figure 7a, which is considered a crucial benchmark for evaluating practical high–temperature performances. Apparently, *x* = 0.02 sample not only possesses a peak *d*_33_ value but also maintains a wide temperature span (≤240 °C). Also, frequency–dependent *ε*_r_ for poled *x* = 0.02 sample is also measured after different annealing temperatures, as shown in Figure 7b. Typical resonance and anti–resonance peaks are observed for sufficiently poled samples. As the temperature increases, the peak gradually degrades, indicating depolarization steadily occurs [48]. These peaks dampen gradually and finally vanish as the temperature increased to~240 °C. This is also indicated by the contour plot, as indicated in Figure 7c. To investigate the depolarization mechanism on the local structure of representative *x* = 0.02 and 0.03 ceramics, in situ Raman spectra is performed, as shown in Figure 7d–g. All Raman spectra are corrected by equation of $I_{c}\left(\omega \right)=I_{m}\left(\omega \right)/\left[n\left(\omega ,T\right)+1\right]$, where *I*_m_(*ω*) is Raman intensity and $n\left(\omega ,T\right)=1/\left[\exp\left(\frac{\hbar \omega }{kT}-1\right)\right]\;$is Bose–Einstein temperature factor [49]. The evolution of wavenumber and FWHM of selected D and G modes are plotted in Figure 7e,f,h,i. The corresponding discontinuities are highlighted by the shadow. The discontinuity occurs around *T*_d_~240 °C and 140 °C for *x* = 0.02 and 0.03 samples, respectively, which indicates an FR phase transition and also accords with the *T*_d_ [50]. 

**Figure 8:**
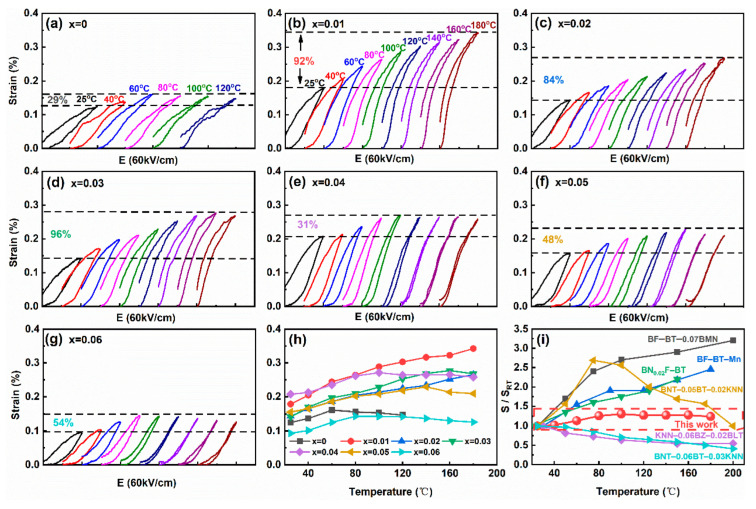
(**a**–**g**) Temperature dependent unipolar *S*–*E* curves at *E* = 60 kV/cm for BF–BT–*x*LNT ceramics; (**h**) evolution of unipolar *S*–*E* with temperature; (**i**) comparison of electrostrain thermal stability between this work with some representative BF–[22,52,53], BNT–[54], and KNN–[55] based ceramics.

Temperature dependent unipolar *S*–*E* curves of BF–BT–*x*LNT ceramics are shown in Figure 8a–g. For *x* ≤ 0.02 compositions with normal ferroelectric character, they exhibit a positive temperature dependence of electrostrain, which mainly originates from thermally activated domain switching [30,51]. For *x* = 0.03 composition, the electrostrain shows a positive temperature dependence below *T*_d_~140 °C, and finally the electrostrain performance remains stable. For *x* = 0.04 sample with less concentration of ferroelectric domains (*R*/*Pc* = 0.25/0.75), the electrostrain shows better temperature stability, which is probably due to a compromise of thermally activated domain switching and agitation. As LNT content further increases, the temperature stability of electrostrain is maintained but strain value decreases. Here, the change rate of electrostrain with temperature is defined as $\eta =\frac{S_{\max}^{'}-S_{\mathrm{RT}}}{S_{\mathrm{RT}}}\times 100\%$, where $S_{\max}^{'}$ is unipolar electrostrain peak value in the test temperature range of 25–180 °C, S_RT_ is the electrostrain at RT. Evolution of unipolar *S*–*E* with temperature for *x* = 0~0.06 compositions are summarized in Figure 8h. Obviously, the *x* = 0.04 component exhibits a large electrostrain and strong temperature stability (*E* = 60 kV/cm). Within 25–180 °C, *x* = 0.04 sample presents a smallest *η* value of 31%, indicating an excellent temperature stability of electrostrain performance. A comparison of electrostrain performance with some representative lead–free piezoelectrics are present in Figure 8i. The electrostrain value fluctuates more than 100% for some BF–based ceramics, showing poor temperature stability [22,52,53]. Also, the electrostrain change exhibits a parabolic–like or monotonous decrease for BNT– and KNN–based ceramics, depending on phase transition temperature [54,55]. The enhanced temperature stability of electrostrain performance for *x* = 0.04 composition is due to the synergistic effect of negative temperature dependent electrostrain for relaxors with *Pc* phase and the positive one for ferroelectric matrix with *R* phase, which strongly suggest the special proportion of relaxor and ferroelectric phase can produce thermally stable electrostrain performance and thus is highly desirable for high–temperature of actuator applications.

**Figure 9:**
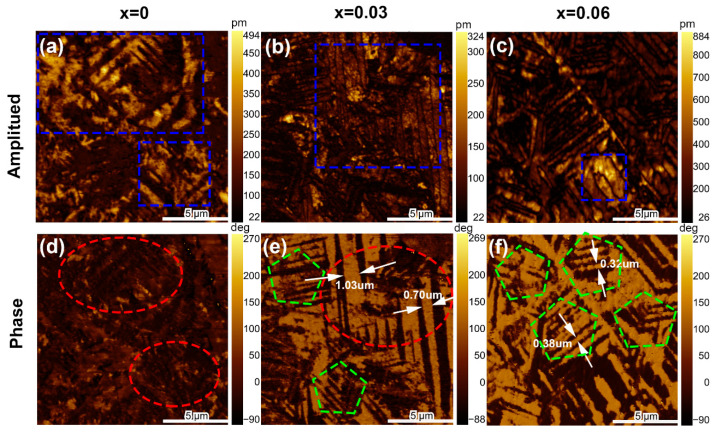
(**a**–**c**) PFM amplitude and (**d**–**f**) phase images for selected *x* = 0, 0.03, 0.06 samples, respectively.

It is well known that evolution in macroscopic performance is accompanied by changes in domain structure, and PFM images can convey domain structure information at a local scale. Figure 9a–c shows the PFM amplitude images for *x* = 0, 0.03, and 0.06 samples, respectively. The highlighted area in the amplitude image represents the piezoelectric response strength [56,57]. Consistent with the piezoelectric performance, *x* = 0 and 0.03 samples exhibit large bright areas, whereas *x* = 0.06 has only few bright areas, as indicated by the blue dashed box. Also, as shown in phase images of Figure 9d–f, broad strip–like long–range ordered macrodomains are clearly observed in the *x* = 0 and 0.03, and thin strip–shaped short–range ordered nanodomains are observed in the dim area of *x* = 0.03 and 0.06. This indicates that both large–sized macrodomains and small–sized nanodomains coexist in the ferroelectric and nonergodic phase composition (*x* = 0.03 as an example). For relaxor phase in *x* = 0.06, small–sized nanodomains occupy almost the entire area. Therefore, large–sized macrodomains with nanodomains blending have a positive effect on the piezoelectric effect, whereas the composition with increased concentration of nanodomains are beneficial for temperature–stable electrostrain performance.

## 4. Conclusions

BF–BT–*x*LNT (*x* = 0~0.06) ceramics are obtained via conventional solid-state synthesis. Rietveld refinement, Raman spectroscopy, and dielectric analysis show the system undergoes from *R*–to–*Pc* phase transition that is driven by LNT addition. Dielectric and impedance spectra show that the addition of LNT reduces the $V_{o}^{¨}$ concentration and greatly increases the resistivity of BF–BT ceramics. At the same time, the piezoelectric performance is optimized at *x* = 0.02 composition (*d*_33_ = 97 pC/N), which is contributed by phase boundary effect and enhanced resistivity for efficiently poling. Notably, temperature stability of electrostrain is obtained for *x* = 0.04 composition, which is due to the synergistic effect of negative temperature dependent electrostrain for relaxor nanodomains with *Pc* phase and the positive one for ferroelectric bulk domains with *R* phase. This is certified by PFM images that nanodomains emerges from ferroelectric matrix with increasing doping content. It can be seen that the addition of LNT not only improves the resistivity and piezoelectric properties of BF–BT ceramics, but also enhances the temperature stability of electrostrain. Therefore, this work has implications for the design and application of high–performance piezoelectrics and temperature–stable electrostrain materials.

## Figures and Tables

**Table 1:** Refined structural parameters and *R*–factors for BF–BT–*x*LNT ceramics.

*x*	Space Group	Lattice Parameters	*R_wp_* (%)	*R_p_* (%)	*χ* ^2^
0	*R3cH* *Pm*$\bar{3}$m	a = b = 5.69552 Å, c = 13.97396 Å, α = β = 90°, γ = 120° a = b = c = 4.02774 Å, α = β = γ = 90°	5.70	4.52	1.31
0.01	*R*3*cH* *Pm*$\bar{3}$*m*	a = b = 5.68390 Å, c = 13.99490 Å, α = β = 90°, γ = 120° a = b = c = 4.02816 Å, α = β = γ = 90°	6.07	4.63	1.67
0.02	*R*3*cH* *Pm*$\bar{3}$*m*	a = b = 5.69602 Å, c = 13.97944 Å, α = β = 90°, γ = 120° a = b = c = 4.02837 Å, α = β = γ = 90°	6.37	4.81	1.56
0.03	*R*3*cH* *Pm*$\bar{3}$*m*	a = b = 5.68895 Å, c = 13.96117 Å, α = β = 90°, γ = 120° a = b = c = 4.02695 Å, α = β = γ = 90°	5.95	4.45	1.47
0.04	*R*3*cH* *Pm*$\bar{3}$*m*	a = b = 5.69720 Å, c = 13.90696 Å, α = β = 90°, γ = 120° a = b = c = 4.02521 Å, α = β = γ = 90°	6.13	4.59	1.64
0.05	*Pm* $\bar{3}$ *m*	a = b = c = 4.02458 Å, α = β = γ = 90°	7.61	5.50	2.44
0.06	*Pm* $\bar{3}$ *m*	a = b = c = 4.02355 Å, α = β = γ = 90°	7.44	5.30	2.21

**Table 2:** Resistance value (Ω) of *R*_b_ at different temperatures obtained by fitting circuit for BF–BT–*x*LNT ceramics.

*x/T*	280 °C	300 °C	320 °C	340 °C	360 °C	380 °C
0	3230	1721	915	414	149	61
0.01	72,374	31,546	14,720	6758	3391	1753
0.02	177,480	84,263	34,951	15,035	7447	3804
0.03	72,973	37,544	19,766	10,816	6113	3675
0.04	61,701	30,854	16,371	8958	5072	2934
0.05	53,899	27,061	14,361	7946	4521	2632
0.06	54,680	28,263	14,881	8208	4689	2739

## Data Availability

The data presented in this study are available on request from the corresponding author.
